# Short-term effects of sildenafil in the treatment of dogs with pulmonary hypertension secondary to degenerative mitral valve disease

**DOI:** 10.14202/vetworld.2020.2260-2268

**Published:** 2020-10-29

**Authors:** Karun Saetang, Sirilak Disatian Surachetpong

**Affiliations:** Department of Veterinary Medicine, Faculty of Veterinary Science, Chulalongkorn University, Bangkok, Thailand

**Keywords:** degenerative mitral valve disease, dogs, pulmonary hypertension, sildenafil

## Abstract

**Background and Aim:**

Pulmonary hypertension (PH) is a common complication of degenerative mitral valve disease (DMVD). Sildenafil, a phosphodiesterase-5 inhibitor, has effects in reducing pulmonary arterial pressure by selectively dilating pulmonary vessels. The study aimed to evaluate the effects of sildenafil in combination with conventional therapy in dogs with PH caused by DMVD.

**Materials and Methods:**

Fourteen dogs diagnosed with PH secondary to DMVD Stage C on conventional therapy were randomly assigned to placebo (n=7) and sildenafil (n=7) groups. On day 0, the recruited dogs underwent physical examinations, clinical score assessments, electrocardiography, systolic blood pressure measurements, blood collection, thoracic radiography, and echocardiography for baseline. The dogs then received a combination of conventional therapy with sildenafil or placebo every 8 h for 1 week. On day 7, all dogs underwent the baseline evaluations again.

**Results:**

The sildenafil group experienced a significant decrease in estimated systolic pulmonary artery pressure (sPAP) (p=0.043) from day 0 to day 7. Moreover, the total clinical scores were decreased in dogs treated with sildenafil relative to those who received the placebo (p=0.007); however, the lung scores were not different between before and after treatment with sildenafil.

**Conclusion:**

Sildenafil had a synergistic effect with conventional therapy in reducing the estimated sPAP and clinical scores in dogs with PH secondary to DMVD.

## Introduction

Degenerative mitral valve disease (DMVD) is the most common acquired cardiovascular disease in small and toy breed dogs [1]. Pulmonary hypertension (PH) is a common complication in dogs affected by DMVD that can worsen the clinical signs and outcome [2,3]. PH involves a persistent increase in the systolic pulmonary artery pressure (sPAP) of more than 30 mmHg. The prevalence of dogs with DMVD that develop PH ranges from 14% to 53% [4].

At present, the standard protocol for the treatment of PH in dogs with DMVD remains uncertain. Although conventional therapies for DMVD, including pimobendan, furosemide, and angiotensin-converting enzyme inhibitors (ACEis), can reduce the sPAP to approximately 20 mmHg [5], additional methods are required for the treatment of PH secondary to DMVD to further decrease the sPAP. Other drugs that have efficacy in decreasing the sPAP include platelet-derived growth factor inhibitors, prostacyclin analogs, endothelin antagonists, and phosphodiesterase-5 inhibitors (PDE-5is) [4,6-8]. PDE-5 is abundantly found in the pulmonary vessels and PDE-5is, such as sildenafil, have a selective vasodilation effect on these pulmonary vessels [4]. The previous studies of PH caused by respiratory and cardiovascular diseases in dogs and rats found that sildenafil can decrease the sPAP [7,9-11]. However, the American College of Veterinary Medicine (ACVIM) does not recommend using sildenafil in dogs with PH secondary to left-sided heart disease because it may increase blood flow to the pulmonary system and worsen the pulmonary edema [12]. At this time, though, no clinical trial exists to support this recommendation.

As mentioned above, dogs affected by DMVD have a high prevalence of the development of PH that can worsen cardiac function, clinical signs, and the median survival time. Furthermore, conventional therapy options to reduce the sPAP are limited. Therefore, additional drugs with selective pulmonary vasodilation effects like sildenafil may improve the outcome. We hypothesized that the combined effect of conventional therapy and sildenafil could help to improve clinical signs and radiographic findings and to reduce the sPAP in dogs with DMVD and PH.

This study aimed to evaluate the effects of sildenafil in combination with conventional therapy on clinical scores, lung scores as assessed by radiography, echocardiographic values, and the estimated sPAP as assessed by echocardiography.

## Materials and Methods

### Ethical approval

The design of the study was a single-blinded, prospective, randomized, and placebo-control study. The owners were informed of the process and signed a consent form. The protocol of this study was approved by the Chulalongkorn University Animal Care and Use Committee (Protocol No. 1831077).

### Study location and period

The study was performed at the Small Animal Teaching Hospital, Faculty of Veterinary Science, Chulalongkorn University, Thailand, from March 2019 to March 2020.

### Inclusion criteria

Dogs with PH caused by DMVD Stage C were recruited to the study. The inclusion criteria were: (1) Small-breed dogs weighing <15 kg and aged older than 6 years; (2) PH secondary to DMVD determined by echocardiographic evidence of a tricuspid regurgitant flow velocity of >2.7 m/s or an estimated sPAP of >30 mmHg [4]; (3) DMVD Stage C defined by radiographic evidence of current or history of pulmonary edema and cardiomegaly (vertebral heart score [VHS] >10.7, [13]) and echocardiographic evidence of mitral valve thickening, mitral valve regurgitation, left atrial enlargement (ratio of the left atrial to aorta diameter [LA:Ao] > 1.5 as measured in the short-axis view [14]), or left ventricular enlargement (left ventricular internal diastolic diameter > 1.7 [15]); and (4) being on conventional therapy consisting of furosemide, pimobendan, and ACEi medication.

### Exclusion criteria

Dogs were excluded from this study for several conditions, including: (1) Other acquired or congenital cardiac diseases or other systemic diseases such as kidney, liver, or gastrointestinal disease; (2) respiratory diseases determined based on history, physical examination, and radiographic findings; and (3) positive heartworm antigen test results (Snap 4Dx; IDEXX, Westbrook, ME, USA). The exclusion criteria also encompassed (4) creatinine level >1.4 mg/dL, alkaline phosphatase (Normal range 10-150 IU/L) or alanine aminotransferase (Normal range 5-60 IU/L) level >3 times the upper limit, and abnormalities in other blood profile values (e.g., red blood cell count [5.5-8.5×10^6^/mm^3^], white blood cell [6-17×10^3^/mm^3^], platelet [200-500×10^3^/mm^3^], albumin [2.6-4.3 g/dL], and total protein [5.1-7.8 g/dL] [16]). Moreover, (5) blood pressure of <100 mmHg or >180 mmHg [17] and (6) a requirement for cardiovascular drugs besides conventional therapy, that is, furosemide, pimobendan, and ACEi medication were selected as exclusion criteria.

### Sample size

This study recruited seven dogs for each group. The sample size was calculated by using a formula, that is, sample size=
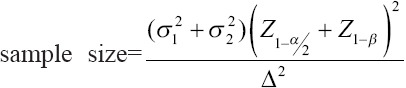
[18]. The mean and standard deviation values were taken from a recent study that investigated the effects of sildenafil in dogs with PH caused by acute pulmonary embolism [19]. The sample size was calculated at 5% for the level of significance and 80% for the power of the study.

### Dogs

On day 0, all recruited dogs underwent physical examinations, clinical score assessments, systemic blood pressure measurements, electrocardiography, blood collection, thoracic radiography, and echocardiography performed by an investigator. The dog information, including age, breed, and sex, was recorded. This study was designed as a single-blinded study. Besides conventional therapy (e.g., ACEis, furosemide, and pimobendan), the owners were blinded to the type of treatment the drug received (i.e., sildenafil or placebo); both sildenafil and placebo were prescribed without labels. Fourteen recruited dogs were randomly divided into the sildenafil and placebo groups at a 1:1 ratio to maintain a similar sample size in both groups. The sildenafil group received a combination of sildenafil (1-3 mg/kg 3 times daily) and conventional therapy. The placebo group received a combination of the placebo (3 times daily) and conventional therapy. Both groups received their respective treatments for 7 days; then, on day 7, the dogs again underwent physical examinations, electrocardiography, systolic blood pressure measurements, blood collection, clinical score assessments, thoracic radiography, and echocardiography performed by the same investigator as before.

### Clinical evaluation

Physical findings, including body condition score, weight, heart rate, heart sounds, respiratory rate, lung sounds, and respiratory patterns were recorded. Electrocardiography was performed in the right lateral recumbency position for 3 min. The systolic blood pressure, using a Doppler device on the median artery of the thoracic limb in the lateral recumbency position, was measured. Each owner was interviewed on day 0 (before treatment) and day 7 (after treatment) by an investigator. The clinical scores included coughing, exercise intolerance, dyspnea, syncope, and appetite (Table-1).

**Table 1:** Scoring protocol for clinical variables.

Variable	Score	Clinical presentation
Cough	1	None
	2	Few times a week
	3	Few times a day
	4	Frequently during the day
Exercise intolerance	1	Dogs had ability to fully exercise
	2	Dog was active. Ability to run was reduced
	3	Dogs were less active than normal. Avoided long walk
	4	Dogs were inactive and only got up to eat, drink, urinate, or defecate
Dyspnea	1	Dogs were able to rest. Resting respiratory rate <25 tpm
	2	Dogs were able to rest. Resting respiratory rate >25 tpm
	3	Dogs were restlessness and had respiratory effort
Syncope	1	None
	2	2-6 times/week
	3	Every day, <3 times/day
	4	>3 times/day
Appetite	1	Increased
	2	Normal
	3	Decreased (>2/3 of normal)
	4	Markedly decreased (<2/3 of normal)

Modified from (Brown *et al*., [7] Arita *et al*., [8]), tpm=time per minute

#### Thoracic radiography

**Figure-1:**
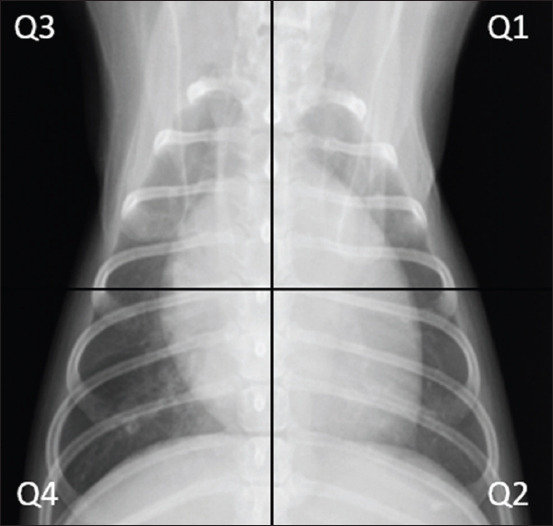
The thoracic radiograph was divided into four quadrants. The vertical line was divided at the middle of the trachea at the thoracic inlet. The horizontal line was perpendicular to the vertical line at the level of the center of the carina.

The right lateral and ventrodorsal views of thoracic radiography were collected. The radiographic examination consisted of lung patterns and the vertebral heart scale [20]. The lung score was evaluated using a method described in a previous study [21]. The lung fields were divided into four quadrants, as seen in Figure-1, and a score was given to each quadrant, where zero points indicated no lung infiltration and one, two, three, and four point(s) indicated lung infiltration of <25%, 25% to 50%, 50% to 75%, and more than 75%, respectively.

### Echocardiography

The echocardiographic values were obtained using an ultrasound machine (Mindray; M9, Shenzhen, China) with 2- to 4- and 4- to 12-MHz phased-array transducers. The echocardiographic examination was performed with the dog in an unsedated condition. M-mode echocardiography was performed in the right parasternal long-axis view [22] and values included the left ventricular internal diastolic diameter, left ventricular internal systolic diameter, left ventricular free-wall thickness during diastole, left ventricular free-wall thickness during systole, ventricular septal thickness during diastole, and ventricular septal thickness during systole. LA:Ao was determined in the right parasternal short-axis views at the mitral valve level [23].

Peak tricuspid regurgitant flow velocity was achieved by measuring the velocity of regurgitant flow at the tricuspid valve during systole in the left apical four-chamber view by spectral flow Doppler echocardiography. In this context, the spectral Doppler cursor aligns parallel to the tricuspid regurgitant flow direction. The velocity was converted to the estimated sPAP by the modified Bernoulli equation (pressure gradient=4 times the peak of tricuspid regurgitant velocity^2^) [4,22].

The ejection time (ET) was obtained by measuring the time at the start to the end of the pulmonary artery flow profile received from the right parasternal short axis at the pulmonic valve level. Separately, the acceleration time (AT) was discerned by measuring the time from the start to the peak of the pulmonary artery flow profile. Then, the ratio of AT to ET (AT:ET) was calculated [23].

The right ventricular ET (RVET), isovolumic relaxation time (IVRT), and isovolumic contraction time (IVCT) were measured by tissue Doppler echocardiography in the left apical four-chamber view [24]. The Tei index was calculated using the equation Tei index=(IVCT+IVRT)/RVET [25].

Tricuspid annular plane systolic excursion to the aortic diameter ratio (TAPSE:Ao) was achieved by M-mode echocardiography in the left apical four-chamber view. The movement of the lateral aspect of the tricuspid annulus was recorded. Furthermore, the distance of the lateral aspect of the tricuspid annulus movement between systole and diastole was measured [26].

The right pulmonary artery distensibility index (RPAD) was obtained by two-dimensional echocardiography. The minimum diastolic (RPA_D_) and maximum systolic (RPA_S_) internal diameters of the right pulmonary artery were measured in the right parasternal short-axis view at the same location. The equation used here was RPAD index=(RPAS−RPAD)/RPAS×100 [27].

### Statistical analysis

Data collected on day 0 and day 7 were analyzed with a statistical software program (SPSS Statistics version 22; IBM Corporation, Armonk, NY, USA). The data distribution was tested using the Kolmogorov–Smirnov test. All data are presented as medians and interquartile ranges (IQRs). Comparing data between groups were achieved with the Mann–Whitney U-test, while the comparison of data over time (between days 0 and 7) was done using the Wilcoxon signed-rank test. For all analyses, p<0.05 was considered to be statistically significant. The relationship between the sPAP and echocardiographic values was assessed using Spearman’s correlation.

## Results

The dog information in the sildenafil and placebo groups on day 0 was similar and is presented in Table-2. All dogs received triple therapy, including furosemide, pimobendan, and ACEi medication. Dogs in the sildenafil group received a median dose of sildenafil of 1.79 (1.69-2.19) mg/kg every 8 h for 1 week.

**Table 2:** The information of recruited dogs on day 0 in the placebo and sildenafil groups.

Parameter	Placebo	Sildenafil
Number of dogs	7	7
Breed	2 Poodles,	2 Poodles,
	2 Chihuahuas, 1	3 Chihuahuas,
	Miniature Pinscher,	1 Pommeranian
	1 Jack Russel, and	1 Shih-tzu
	1 Shih-tzu	
Sex	4 Males, 3 Females	3 Males, 4 Females
Age (years)	12 (9.5-12.5)	11 (10.0-11.5)
Body condition score (0-5)	3.0 (3.0-3.5)	3.0 (2.0-4.0)
Body weight (kg)	5.10 (4.41-5.38)	4.10 (4.01-5.42)

All values presented as median and interquartile range.

The vital signs, lung sounds, respiratory patterns, systemic blood pressure values, and electrocardiography of the placebo and sildenafil groups on days 0 and 7 are shown in Table-3. The vital signs (temperature, heart rate, and respiratory rate) and systemic blood pressure values were not different on either day 0 or day 7 between the sildenafil and placebo groups. In addition, the changes in the vital signs and systemic blood pressure values were not different from day 0 to day 7 between the sildenafil and placebo groups.

**Table 3:** The vital signs, electrocardiography, and systemic blood pressure of in the placebo and sildenafil groups on day 0 and day 7.

Parameter	Placebo day 0	Sildenafil day 0	Placebo day 7	Sildenafil day 7
Temp.(°F)	101	101	100.4	100.0
	(100.5-101.0)	(100.2-101.1)	(100.2-101.0)	(100.0-101.2)
HR (bpm)	137 (122-170)	144 (126-179)	143 (136-168)	150 (112-169)
RR (bpm)	56 (40-66)	42 (29-57)	45 (38-57)	45 (27-60)
Lung sound	Normal (1/7)	Normal (4/7)	Normal (3/7)	Normal (4/7)
	Increased (3/7)	Increased (3/7)	Increased (2/7)	Increased (3/7)
	Crackled (3/7)		Crackled (2/7)	
Respiratory patterns	Normal (2/7)	Normal (2/7)	Normal (4/7)	Normal (3/7)
	Tachypnea (3/7)	Tachypnea (1/7)	Tachypnea (2/7)	Tachypnea (2/7)
	Dyspnea (1/7)	Dyspnea (1/7)	Dyspnea (0/7)	Panting (2/7)
	Panting (1/7)	Panting (3/7)	Panting (1/7)	
SBP (mmHg)	126 (111-143)	133 (123-148)	126 (122-137)	137 (130-156)
ECG	RSA (1/7)	SR (4/7)	SR (4/7)	SR (4/7)
	SR (2/7)	ST (3/7)	ST (3/7)	ST (3/7)
	ST (4/7)			

All values presented as median and IQR. Temp.=Body temperature, RSA=Respiratory sinus arrhythmia, SR=Sinus rhythm, ST=Sinus tachycardia, HR=Heart rate, RR=Respiratory rate, ECG=Electrocardiography, SBP=Systolic blood pressure

The blood profile values of the placebo and sildenafil groups on days 0 and 7 are displayed in Table-4. The blood profile values of the sildenafil and placebo groups were the same on day 0. There was no difference in the changes in the blood profile values that occurred from day 0 to day 7 in the sildenafil and placebo groups. Furthermore, no difference between the sildenafil and placebo groups was found in the same values on either day 0 or day 7.

**Table 4:** The blood profile values of the placebo and sildenafil groups on day 0 and day 7.

Parameter	Placebo day 0	Sildenafil day 0	Placebo day 7	Sildenafil day 7
RBC (10^6^/mm^3^)	6.25 (6.01-6.76)	6.24 (5.70-6.58)	6.67 (6.28-6.78)	6.37 (5.83-6.58)
WBC (10^3^/mm^3^)	12.87 (9.74-13.35)	10.49 (8.11-12.77)	11.17 (9.73-11.82)	8.52 (8.00-11.58)
Platelets (10^3^/mm^3^)	278 (223-435)	398 (356-440)	290 (245-498)	288 (241-428)
Creatinine (mg/dL)	1.00 (0.75-1.20)	1.00 (0.95-1.25)	1.20 (0.79-1.20)	1.10 (0.95-1.10)
BUN (mg/dL)	30.6 (23.95-38.65)	35.40 (29.20-36.90)	31.50 (23.45-45.05)	36.10 (27.35-40.00)
ALT (IU/L)	54 (44-106)	75 (61-109)	53 (49-103)	72 (55-103)
ALP (IU/L)	86 (49-108)	105 (76-293)	89 (47-109)	78 (76-252)
Total protein (g/dL)	6.20 (5.90-6.55)	6.60 (5.90-7.00)	6.30 (6.15-6.75)	6.70 (6.40-7.10)
Albumin (g/dL)	3.20 (2.75-3.40)	3.10 (2.70-3.35)	3.30 (2.95-3.40)	3.20 (2.80-3.40)
Globulin (g/dL)	3.20 (2.80-3.35)	3.20 (2.95-3.60)	3.30 (3.10-3.35)	3.60 (3.20-3.75)

All values presented as median and interquartile range, RBC=Red blood cell, WBC=White blood cell, BUN=Blood urea nitrogen, ALT=Alanine aminotransferase, ALP=Alkaline phosphatase

The VHS and lung score of the placebo and sildenafil groups on days 0 and 7 are shown in Table-5. Notably, the thoracic radiography of all dogs revealed pulmonary edema and cardiomegaly. Moreover, the lung scores of quadrants 1 and 3 of the sildenafil group on day 0 were significantly lower than those in the placebo group (p=0.024 and 0.024, respectively). The average lung score of the sildenafil group on day 7 tended to be increased but not significantly (p=0.157).

**Table 5:** The vertebral heart score and lung score of the placebo and sildenafil groups on day 0 and day 7.

Parameter	Placebo day 0	Sildenafil day 0	Placebo day 7	Sildenafil day 7
VHS	11.50 (11.35-12.60)	11.70 (11.50-12.75)	11.70 (11.20-12.60)	11.80 (11.60-12.85)
Q1	1.0 (0.0-1.0)*	0*	1.0 (0.0-1.5)	0
Q2	1.0 (1.0-2.0)	1.0 (1.0-1.5)	1.0 (1.0-2.0)	2.0 (1.0-2.0)
Q3	1.0 (0.0-1.0)*	0*	1.0 (0.0-1.5)	0
Q4	1.0 (1.0-1.5)	1.0 (1.0-1.5)	2.0 (1.0-2.0)	2.0 (1.0-2.0)
Average LS	1.0 (0.5-1.5)	0.5 (0.5-0.63)	1.0 (0.5-1.88)	0.75 (0.5-0.75)

* p<0.05, Compared between placebo and sildenafil on day 0. All values presented as median and interquartile range. VHS=Vertebral heart score, Q1=Quadrant 1, Q2=Quadrant 2, Q3=Quadrant 3, Q4=Quadrant 4, LS=Lung score

The clinical scores of the placebo and sildenafil groups on days 0 and 7 are included in Table-6. Thirteen dogs (92%) presented with coughing, seven (50%) presented with exercise intolerance, 2 (14%) presented with dyspnea, 1 (7%) presented with inappetence, and none presented with syncope. There was no difference in clinical scores between the sildenafil and placebo groups from day 0 to day 7. Moreover, the clinical scores of each parameter did not vary significantly between the sildenafil and placebo groups on day 0 and day 7. However, the total clinical score was significantly different between both groups on day 7 (p=0.007).

**Table 6:** The clinical scores of the placebo and sildenafil groups on day 0 and day 7.

Parameter	Placebo day 0	Sildenafil day 0	Placebo day 7	Sildenafil day 7
Inappetite^1^	2.0 (2.0-2.0)	2.0 (2.0-2.0)	2.0 (2.0-2.0)	2.0 (2.0-2.0)
Exercise intolerance^2^	2.0 (1.0-2.5)	1.0 (1.0-2.0)	2.0 (1.5-2.5)	1.0 (1.0-2.0)
Coughing^3^	3.0 (2.5-3.0)	3.0 (2.5-3.0)	3.0 (2.0-3.5)	2.0 (1.5-3.0)
Dyspnea^1^	1.0 (1.0-1.0)	1.0 (1.0-1.0)	1.0 (1.0-1.5)	1.0 (1.0-1.0)
Syncope^5^	1.0 (1.0-1.0)	1.0 (1.0-1.0)	1.0 (1.0-1.0)	1.0 (1.0-1.0)
Total score	4.0 (3.0-4.5)	3.0 (3.0-3.5)	4.0 (3.0-4.5)	2.0 (2.0-3.0)

1 Inappetite score – 1=Increased appetite, 2=Normal, 3=Can eat >2/3 of normal, 4=Can eat <2/3 of normal.

2 Exercise intolerance score – 1=None, 2=Dog is active, Ability to run is reduced, 3=Dogs is less active than normal, avoid long walk, 4=Dogs is inactive and only get up to eat, drink, urinate, or defecate.

3 Coughing score – 1=None, 2=Few times a week, 3=Few times a day, 4=Frequently during the day.

4 Dyspnea – 1=Dogs is able to rest, resting respiratory rate <25 times/min, 2=Dogs is able to rest, resting respiratory rate >25 times/min, 3=Dogs is restlessness, respiratory effort.

5 Syncope – 1=None, 2=2-6 times/week, 3=Everyday, <3 times/day, 4=Severe >3 times/day. All values presented as median and interquartile range.

The echocardiographic values are shown in Table-7. All dogs had an intermediate to high probability of PH based on the 2020 classification scheme of the ACVIM [12]. On day 0, the interventricular septal thickness during systole in the placebo group was greater than that in the sildenafil group; however, the value was still within the normal range (0.43-0.79) (p=0.047). On day 7, the LA and LA:Ao values of the placebo group were significantly decreased when compared to those recorded on day 0 (p=0.028 and 0.018, respectively). Conversely, the LA and LA:Ao values of the sildenafil group were not different on day 0 and day 7. On day 7, the tricuspid regurgitant flow velocity and the estimated sPAP of the sildenafil group were significantly decreased when compared with those measured on day 0 (p=0.043 and 0.043, respectively); notably, the estimated sPAP was decreased by approximately 7 mmHg. The other echocardiographic parameters were also different between before and after treatment with sildenafil. In addition, the sPAP was positively correlated with the heart rate (r=0.70; p=0.005) and IVCT (r=0.63; p=0.014) (Table-8).

**Table 7:** The echocardiographic values of the placebo and sildenafil groups on day 0 and day 7.

Parameter	Placebo day 0	Sildenafil day 0	Placebo day 7	Sildenafil day 7
VSd	0.44 (0.41-0.50)	0.43 (0.40-0.49)	0.43 (0.37-0.49)	0.48 (0.40-0.51)
LVIDd	1.99 (1.89-2.09)	1.92 (1.79-2.04)	1.96 (1.77-2.04)	1.87 (1.74-2.05)
LVWd	0.34 (0.33-0.42)	0.34 (0.32-0.38)	0.41 (0.37-0.47)	0.37 (0.35-0.40)
VSs	0.76+ (0.66-0.77)	0.62+ (0.58-0.66)	0.67 (0.59-0.80)	0.62 (0.58-0.68)
LVIDs	0.98 (0.83-1.04)	1.06 (0.82-1.19)	1.02 (0.91-1.06)	1.08 (0.66-1.16)
LVWs	0.62 (0.58-0.73)	0.69 (0.56-0.76)	0.60 (0.58-0.68)	0.71 (0.58-0.78)
LA	1.68* (1.43-1.82)	1.57 (1.46-1.93)	1.50* (1.15-1.68)	1.63 (1.49-1.80)
Ao	0.63 (0.59-0.69)	0.66 (0.58-0.81)	0.63 (0.62-0.75)	0.76 (0.61-0.79)
LA:Ao	2.40* (2.29-2.61)	2.66 (2.13-2.74)	2.05* (1.97-2.12)	2.23 (2.06-2.51)
FS (%)	51 (47-57)	48 (41-54)	48 (42-54)	48 (43-60)
RPAD (%)	29 (24-33)	21 (13-25)	19 (13-24)	15 (9-16)
TR (m/s)	3.28 (3.15-3.56)	3.69# (3.30-3.98)	3.51 (3.10-3.62)	3.43# (3.20-3.68)
sPAP (mmHg)	43.03 (39.79-50.98)	54.46# (43.66-63.30)	49.51 (38.60-52.46)	47.13# (41.08-54.15)
AT (msec)	47 (39-56)	44 (31-61)	47 (36-52)	50 (47-61)
ET (msec)	111 (92-121)	97 (78-127)	108 (81-120)	126 (98-142)
AT:ET	0.48 (0.41-0.53)	0.46 (0.41-0.50)	0.44 (0.43-0.46)	0.46 (0.42-0.51)
IVCT(msec)	41 (40-55)	53 (42-56)	43 (38-48)	50 (32-76)
IVRT(msec)	52 (39-61)	39 (33-56)	56 (46-66)	61 (54-72)
RVET(msec)	117 (88-143)	117 (110-126)	116 (97-131)	131 (118-136)
Tei index	0.74 (0.68-1.19)	0.76 (0.69-0.80)	0.79 (0.79-0.97)	0.78 (0.58-1.02)
TAPSE:Ao	1.11 (0.74-1.23)	1.27 (0.99-1.39)	1.27 (1.13-1.40)	0.97 (0.93-1.24)

+ p<0.05, compared between placebo and sildenafil on day 0,

* p<0.05, compared between day 0 and day 7 of placebo group,

# p<0.05, compared between day 0 and day 7 of sildenafil group. All values presented as median and interquartile range. VSd=Ventricular septal thickness during diastole, LVIDd=Left ventricular internal diastolic diameter, LVWd=Left ventricular free wall thickness during diastole, VSs=Ventricular septal thickness during systole, LVIDs=Left ventricular internal systolic diameter, LVWs=Left ventricular free wall thickness during systole, LA=Left atrial diameter, Ao=Aortic diameter, LA:Ao=Ratio of left atrium to aorta, FS=Fraction shortening, RPAD=Right pulmonary artery distensibility index, TR=Tricuspid regurgitant flow velocity, sPAP=Systolic pulmonary artery pressure, AT=Acceleration time, ET=Ejection time, AT:ET=Ratio of acceleration time to ejection time, IVCT=Isovolumetric contraction time, IVRT=Isovolumetric relaxation time, RVET=Right ventricular ejection time, TAPSE:Ao=Ratio of tricuspid annular plane systolic excursion to aortic diameter

**Table 8:** The correlation between estimated systolic pulmonary artery pressure and parameters of echocardiography.

Parameter	r	p-value
HR	0.70*	0.005
VSd	0.15	0.593
LVIDd	−0.30	0.284
LVWd	−0.03	0.904
VSs	0.20	0.477
LVIDs	−0.20	0.482
LVWs	0.002	0.994
LA	0.20	0.473
Ao	0.06	0.834
LA:Ao	0.12	0.675
FS	0.14	0.626
RPAD	0.12	0.675
AT	−0.13	0.658
ET	0.07	0.805
AT:ET	−0.34	0.226
IVCT	0.63*	0.014
IVRT	−0.33	0.242
RVET	−0.53	0.050
Tei index	0.43	0.124
TAPSE:Ao	−0.20	0.480

* p<0.05, VSd=Ventricular septal thickness during diastole, LVIDd=Left ventricular internal diastolic diameter, LVWd=Left ventricular free wall thickness during diastole, VSs=Ventricular septal thickness during systole, LVIDs=Left ventricular internal systolic diameter, LVWs=Left ventricular free wall thickness during systole, LA=Left atrial diameter, Ao=Aortic diameter, LA:Ao=Ratio of left atrium to aorta, RPAD=Right pulmonary artery distensibility index, TR=Tricuspid regurgitant flow velocity, sPAP=Systolic pulmonary artery pressure, AT=Acceleration time, ET=Ejection time, AT:ET=Ratio of acceleration time to ejection time, IVCT=Isovolumetric contraction time, IVRT=Isovolumetric relaxation time, RVET=Right ventricular ejection time, TAPSE:Ao=Ratio of tricuspid annular plane systolic excursion to aortic diameter

## Discussion

This study demonstrates the effects of sildenafil in combination with conventional therapy on the clinical scores, radiographic lung scores, echocardiographic values, and estimated sPAP values of dogs affected with PH secondary to DMVD.

The previous studies have reported that sildenafil may improve clinical scores in dogs with PH secondary to respiratory disease [4,21,28]. A similar result was also found in the present study, in that the clinical scores were significantly decreased after treatment with sildenafil when compared with those scores of dogs who received the placebo. Common clinical presentations of DMVD dogs with PH in the present study included coughing, exercise intolerance, dyspnea, and inappetence. None of the dogs in the present study had syncope, which varies from the findings of the previous studies that reported syncope, coughing, and exercise intolerance to be common clinical presentations in dogs with PH [4,11,28].

The dogs in the present study also had a rapid respiratory rate (median: 51 [IQR: 31-65] breaths/min) and pulmonary edema as assessed by radiography on day 0. On day 7, after treatment with sildenafil, the respiratory rate and lung scores were not different from those recorded on day 0. This result is different from a previous study that reported an improvement in lung scores at a median time point of 3.5 days following sildenafil treatment. However, most of the dogs in this prior study were affected by PH secondary to respiratory disease [21]. The average lung score in dogs treated with sildenafil in this study did tend to increase but did not reach statistical significance. This result is consistent with the recommendations of the ACVIM suggesting that sildenafil be prescribed carefully in dogs with pulmonary edema due to left heart disease as this medication carries the potential risk of inducing the progression of pulmonary edema [12]. Specifically, sildenafil dilates the pulmonary artery and reduces the afterload to the heart, resulting in increased blood flow to the pulmonary system, which may contribute to pulmonary edema in left-sided heart disease [4,21].

The left ventricular dimension was not different between before and after treatment with sildenafil for 7 days. This result was similar to that of a previous study demonstrating that long-term treatment (160 days) with sildenafil did not affect the left ventricular dimension in dogs with DMVD stage B [29]. The LA and LA:Ao values in the placebo group were significantly decreased on day 7, while those in the sildenafil group were not different between before and after treatment. A decrease in LA:Ao may indicate a reduction in the degree of blood return from the lungs to the left atrium. A chronic elevation of PAP increases the afterload to the heart, leading to decreased blood flow to the left atrium [30].

On day 0, the recruited dogs showed increased fractional shortening [median: 50% (IQR: 45-58%)]. An observed increase in fractional shortening in dogs with mitral regurgitation may be due to an increase in the preload in the left ventricle [31]. A few studies investigating the effect of sildenafil on fractional shortening have been published to date. An improvement of fractional shortening has been reported in mice with induced myocardial infarction that received sildenafil [32]. Another experiment conducted in rats with induced aortic regurgitation demonstrated that sildenafil inhibited left ventricular remodeling and improved fractional shortening [33]. However, an effect of sildenafil in increasing fractional shortening was not observed in dogs affected by PH secondary to DMVD in the present study. A similar result was also gained in a previous study, which demonstrated that sildenafil did not affect fractional shortening in dogs with DMVD stage B [29].

Further, the present study showed that treatment with sildenafil did not affect the RPAD index. This result is in contrast with a previous study indicated that dogs affected with PH secondary to respiratory disease experienced an increase in the RPAD index after treatment with sildenafil and tadalafil [34].

Our recruited dogs had a shortened AT of the pulmonary artery flow as assessed by spectral Doppler echocardiography (median: 45 [IQR: 33-59] ms). The previous studies have found that the AT and ET were increased after treatment with sildenafil in dogs affected by PH secondary to respiratory and cardiovascular diseases [9,35]. It has also been suggested that an increase in the AT and ET occurs secondary to a decrease in PAP [9,23]. In the present study, the AT and ET were not different between before and after treatment with sildenafil. This may be due to a small reduction in the PAP after treatment with sildenafil that may not be enough to reflect any changes in the pulmonary artery flow profile.

The Tei index, which an index for evaluating global right ventricular myocardial function [22], was increased in dogs with PH secondary to DMVD in the present study. In addition, a previous study reported the existence of both a positive correlation between the right ventricular Tei index and sPAP and survival time in dogs with DMVD [36]. Other research has determined that sildenafil may improve the Tei index in PH-induced rats and human patients with PH secondary to a hypoxia condition [37,38]. However, a difference in the Tei index between before and after treatment with sildenafil in the present study was not observed.

In the present study, the TAPSE:Ao increased but had no relationship with the sPAP. This result is different from that of a previous study reporting that TAPSE:Ao had a negative relationship with the sPAP [39]. The present study also showed that TAPSE:Ao was not different between dogs treated with placebo and sildenafil, which is similar to findings of the previous studies indicating that no improvement in TAPSE in PH-induced rats and human patients with PH treated with sildenafil could be found [38,40].

The estimated sPAP in this study was decreased following treatment with sildenafil. This finding is similar to other research focusing on sildenafil treatment in dogs with PH caused by respiratory and various cardiac diseases [7,28]. Left-sided heart failure causes pulmonary venous hypertension secondary to an increased left atrial pressure and reactive pulmonary arterial vasoconstriction. In addition, the increased thickness of the pulmonary vascular wall or the pulmonary vascular remodeling provoked by an increased PAP can worsen and enhance the progression of PH [4]. Considering these mechanisms, sildenafil may help by decreasing PAP and vascular remodeling.

The present study revealed a positive relationship existed between the heart rate and estimated sPAP, which is similar to that of a previous study demonstrating that dogs with higher PAP values, had higher heart rates [41]. The rationale of this relationship may be as follows: Dogs affected by PH experience a reduction in stroke volume secondary to an increase in afterload; therefore, their heart rate is increased to maintain the cardiac output. A study in human patients affected by pulmonary arterial hypertension showed a significant change in heart rate after a 6-min walk test [42]. In the present study, the estimated sPAP correlated positively with the IVCT, which may have occurred because the heart takes a longer period of time to generate pressure in the isovolumic contraction phase when the PAP is rising [43].

The target pulmonary vasodilating effect of sildenafil decreases the pulmonary pressure while having minimal effects on systemic blood pressure [44]. However, some studies have found that systemic blood pressure tended to decrease – remained within the normal range – after treatment with sildenafil [7,9,28]. The present study showed that systolic blood pressure in dogs affected with PH secondary to DMVD remained unchanged after treatment with sildenafil.

Sildenafil has minimal systemic effects because it has a selective vasodilation effect in organs with abundant PDE-5 expression, such as the lungs [4]. Adverse effects of sildenafil have rarely been reported in dogs [9]. The present study showed no difference existed in complete blood counts or blood chemistry profile values between the placebo and sildenafil groups on day 7 and from day 0 to day 7 in the sildenafil group. In humans, one of the major side effects of sildenafil is cardiac arrhythmia [45]. The presence of cardiac arrhythmia was not found in the present study after treatment with sildenafil. These results suggest that it is safe to use sildenafil, at least for the short-term, in dogs affected by PH secondary to DMVD.

However, there were several limitations to this study. First, this investigation focused on the short-term effects of sildenafil in the treatment of dogs affected by PH secondary to DMVD; thus, the results cannot be extrapolated for use in the long-term treatment and management of dogs with PH from other causes. Second, the PAP was estimated by measuring the tricuspid regurgitant velocity as assessed by echocardiography. This technique may be complicated by several factors affecting the estimated sPAP including the poor resolution of images due to pulmonary pathology, the alignment of the cursor to the tricuspid jet while measuring, and the right ventricular systolic function [9]. Finally, the number of recruited dogs in this study was low.

## Conclusion

Sildenafil boasts a synergistic effect to conventional therapy in reducing the estimated sPAP; however, it does not affect the systemic blood pressure or blood profile values. Sildenafil had no short-term effects on lung lesions assessed by radiography but could help with improving clinical signs of dogs with PH secondary to DMVD Stage C. The long-term effects of sildenafil should be investigated to compile more information regarding optimal use conditions.

## Authors’ Contributions

KS: Performed experiment, data collection, data analysis and writing first draft. SDS: Supervised, performed experiment, data validation, review, and editing. Both authors read and approved the final manuscript.
